# SIRT1 and SIRT2 inhibition impairs pediatric soft tissue sarcoma growth

**DOI:** 10.1038/cddis.2014.385

**Published:** 2014-10-23

**Authors:** L Ma, W Maruwge, A Strambi, P D'Arcy, P Pellegrini, L Kis, A de Milito, S Lain, B Brodin

**Affiliations:** 1 Department of Oncology and Pathology, Karolinska Institutet, Stockholm, Sweden; 2 Department of Microbiology, Tumor and Cell Biology, Karolinska Institutet, Stockholm, Sweden

## Abstract

Sirtuins are NAD+ dependent deacetylases and/or ADP-ribosyl transferases active on histone and non-histone substrates. The first sirtuin was discovered as a transcriptional repressor of the mating-type-loci (*Silent Information Regulator sir2*) in the budding yeast, where it was shown to extend yeast lifespan. Seven mammalian sirtuins (SIRT1-7) have been now identified with distinct subcellular localization, enzymatic activities and substrates. These enzymes regulate cellular processes such as metabolism, cell survival, differentiation, DNA repair and they are implicated in the pathogenesis of solid tumors and leukemias. The purpose of the present study was to investigate the role of sirtuin expression, activity and inhibition in the survival of pediatric sarcoma cell lines.We have analyzed the expression of SIRT1 and SIRT2 in a series of pediatric sarcoma tumor cell lines and normal cells, and we have evaluated the activity of the sirtuin inhibitor and p53 activator tenovin-6 (Tv6) in synovial sarcoma and rhabdomyosarcoma cell lines. We show that SIRT1 is overexpressed in synovial sarcoma biopsies and cell lines in comparison with normal mesenchymal cells. Tv6 induced apoptosis as well as impaired autophagy flux. Using siRNA to knock down SIRT1 and SIRT2, we show that the expression of both proteins is crucial for the survival of rhabdomyosarcoma cells and that the loss of SIRT1 expression results in a decreased LC3II expression. Our results show that SIRT1 and SIRT2 expressions are crucial for the survival of synovial sarcomas and rhabdomyosarcomas, and demonstrate that the pharmacological inhibition of sirtuins impairs the autophagy process and induces tumor cell death.

Sirtuins are a family of deacetylases (class III histone deacetylases, HDACs) and ADP-ribosyl transferases with evolutionary conserved functions in cellular metabolism and chromatin regulation.^1^ The first sirtuin, silent information regulator (SIR2), was identified with a role in silencing the mating-type loci and prolonging lifespan of budding yeast.^2^ Aside from deacetylating histones, sirtuins are also active on non-histone substrates and differ from other histone deacetylases in that their activity is NAD+ dependent. As NAD+ is a key component in energy and signal-transduction pathways, sirtuin's activity is linked to cell metabolism but also with other cellular processes such as cell cycle DNA repair and transcription.

Seven sirtuins (SIRT1-7) have been identified in mammals, all of them sharing the NAD+ binding and catalytic domain, but targeting different substrates and distinct but not exclusive subcellular localizations. Some sirtuins are primarily in the nucleus (SIRT1, 2, 6 and 7), others in the cytoplasm (SIRT2) and a third group locates mostly in mitochondria (SIRT3, 4 5).^3^

SIRT1, 3 and 6 have been implicated in the regulation of glucose and lipid metabolism during cellular stress induced by calorie restriction, probably by sensing intracellular NAD+ levels. Sirtuins are also associated with cancer, as they deacetylate cancer-associated transcription factors. This was first demonstrated for SIRT1 that deacetylates lysine 382 of the p53 tumor suppressor protein (^K382-^p53), a post-translational modification that favors p53 degradation.^4, 5, 6^

SIRT1 is overexpressed in different tumor types such as acute myeloid leukemia, colon and prostate cancers, but this correlation is not found for bladder carcinoma, glioblastoma nor ovarian cancer.^7^ The oncogenic role of SIRT1 is controversial, as this sirtuin is also involved in the maintenance of DNA integrity. SIRT1 relocates at double- and single-strand break sites and deacetylates proteins involved in DNA repair such as Ku70,^8^ and the mutant mice have an impaired DNA damage response and are prone to develop tumors.^9^

Several studies support a role for SIRT2 as tumor suppressor protein. SIRT2 is downregulated in human gliomas^8,10^ and has been implicated in the maintenance of genomic integrity by releasing severely damaged cells from mitotic arrest, forcing them into apoptosis.

In the present study, we have analyzed the expression and activity of SIRT1 and SIRT2 in the most common pediatric soft tissue sarcomas, rhabdomyosarcoma and synovial sarcoma can be of two histological subtypes; the embryonal subtype (ERMS) is genetically characterized by the loss of the short arm of chromosome 11. The alveolar rhabdomyosarcoma is characterized by the chromosomal translocation that fuses the *PAX3* or *PAX7* genes with the gene encoding the transcription factor FOXO1/FKHR.

Synovial sarcoma is the second most frequent soft tissue sarcoma (STS) developing in children and young adults, and is genetically characterized by the translocation t(18;X) and subsequent expression of the *SS18/SSX* fusion gene. These tumors rarely carry inactivating mutations in the *p53* gene, yet, p53 tumor suppressor function is damaged due to the ubiquitination and degradation of p53 induced by SS18/SSX.^11^

In the present paper, we have investigated the expression and activity of SIRT1 and SIRT2 in synovial sarcomas and rhabdomyosarcomas, and we evaluated the effects of sirtuin modulation with the small-molecule tenovin-6 and with SIRT1 and SIRT2 siRNA.

## Results

### SIRT1 is overexpressed in synovial sarcoma tumors and soft tissue sarcoma cell lines

**Figure 1:**
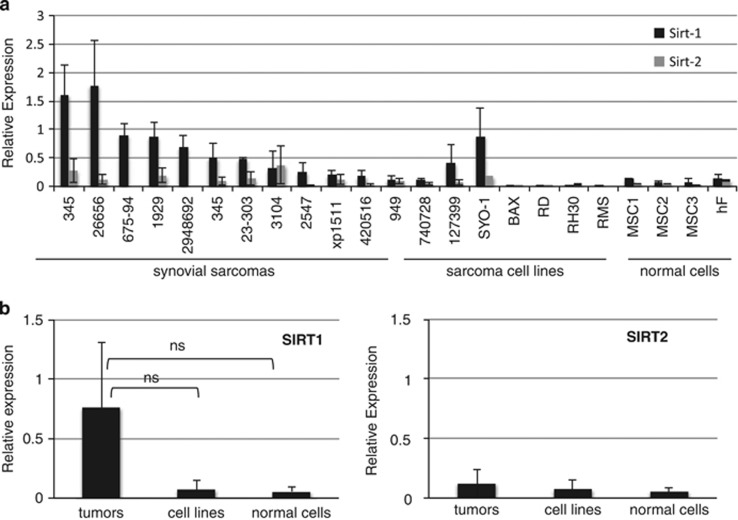
Sirtuin 1 is overexpressed in primary synovial sarcoma tumors compared with normal mesenchymal cells. (**a**) Quantitative expression of SIRT1 and SIRT2 was determined by QRT-PCR in RNA samples obtained from fresh frozen synovial sarcomas, synovial sarcoma cell lines (1273-99, Syo1, Bax) rhabdomyosarcoma cell lines (RD, RMS and RH30), a malignant peripheral nerve sheath tumor (MPNST) cell line (740728), in three different primary mesenchymal stem cells: MSC 1–3 and in human diploid fibroblasts hF. Relative expression denotes *Δ*CT values of SIRT1 or SIRT2 in relation to GAPDH. (**b**) Graphic presentation of SIRT1 and SIRT2 expression in grouped samples. Student's *t*-test showed no statistical difference among the groups (NS)

We quantified SIRT1 and SIRT2 mRNA expression in 12 fresh frozen synovial sarcomas (SS), 7 sarcoma cell lines and 4 primary human mesenchymal cells (diploid fibroblasts and mesenchymal stem cells) using QRT-PCR. SIRT1 was expressed in all SS examined and in the SS cell lines Syo-1 and 1273-99. Overall, SIRT1 expression was higher in tumors than in normal mesenchymal cells, but this difference was not statistically significant (Figure 1b). We did not find differences in SIRT2 expression among the groups, although in general we observed that SIRT2 levels were lower than SIRT1 in most samples (Figures 1a and b). SIRT1 and SIRT2 mRNA expressions in the rhabdomyosarcoma cell lines RD, RH30 and RMS were the lowest among the samples examined.

### Pharmacological inhibition of SIRT1 and SIRT2 with tenovin-6 has antiproliferative effects in pediatric sarcoma cell lines

To investigate a possible role of sirtuins on soft tissue sarcoma growth, we tested the activity of the SIRT1and SIRT2 inhibitor tenovin-6 (Tv6) in seven pediatric soft tissue sarcoma cell lines including four synovial sarcomas (wild-type p53) and three rhabdomyosarcomas (mutated p53 gene). Tv6 treatment inhibited cell proliferation in all sarcoma cell lines tested independent on p53 status with IC_50_ ranging between 1.3 and 5.5 *μ*M at 48 h as determined by using the Wst-1 assay (Table 1 and Supplementary Figure 1).

**Figure 2:**
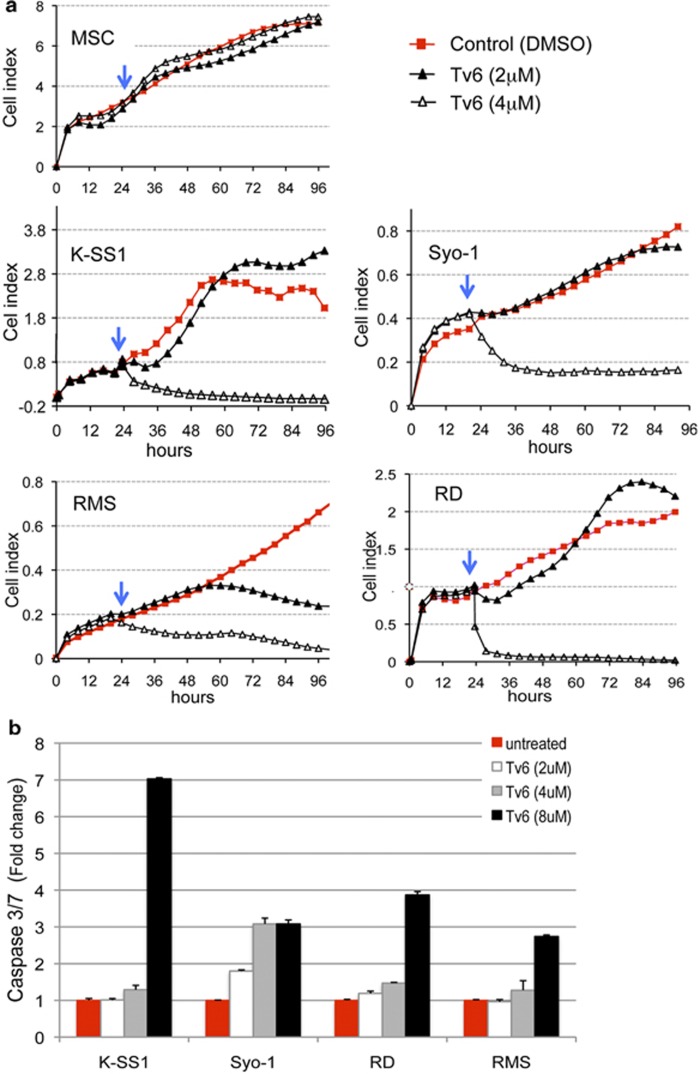
The sirtuin inhibitor tenovin-6 inhibits cell proliferation and induces caspase 3/7 activity in pediatric soft tissue sarcomas. (**a**) Cell proliferation curves of control and tenovin-6 (Tv6)-treated soft tissue sarcoma cell lines followed in real time over a 96-h period using the xCELLigence bioanalyzer. Cells were plated and allowed to form monolayers. The drug was added 24 h after plating (blue arrow). Cell index is a relative unit derived from changes in electrical impedance in a surface; in this case, impedance is proportional to cell confluence giving a quantitative rate of cell numbers. (**b**) Caspase 3/7 activity was measured in cells after 48 h treatment with Tv6. The fold change is relative to untreated control cells (value=1). Syo-1 and K-SS1: synovial sarcoma cell lines. RD: embryonal rhabdomyosarcoma. RMS: alveolar rhabdomyosarcoma. MSC: mesenchymal stem cells

We then followed the activity of Tv6 in real time for 96 h in two synovial sarcomas (K-SS1 and Syo-1) and two rhabdomyosarcoma cell lines (RD and RMS). Figure 2a shows that Tv6 had a rapid and significant cytotoxic effect at 4 *μ*M concentration in the four lines tested without affecting the viability of primary mesenchymal stem cells. It can be also observed that the activity of Tv6 is cell-type and time dependent. For example, the antiproliferative effect of Tv6 at 2 *μ*M concentration was evident in RMS cells at 60 h.

To investigate whether apoptosis was triggered following Tv6 treatment, we quantified the activity of caspases 3 and 7 in treated and control cells. Whereas concentrations above the IC_50_ (8 *μ*M) clearly induced apoptosis, doses within the IC_50_ range only induced slight increments in caspase 3/7 activity in KSS1, RD and RMS cell lines. In the Syo-1 cell line, apoptosis was observed at all concentrations tested. These results show that Tv6 induces apoptosis in a dose-dependent manner (Figure 2b).

### Tenovin-6 impairs sirtuin deacetylating activity without targeting p53 acetylation

**Figure 3:**
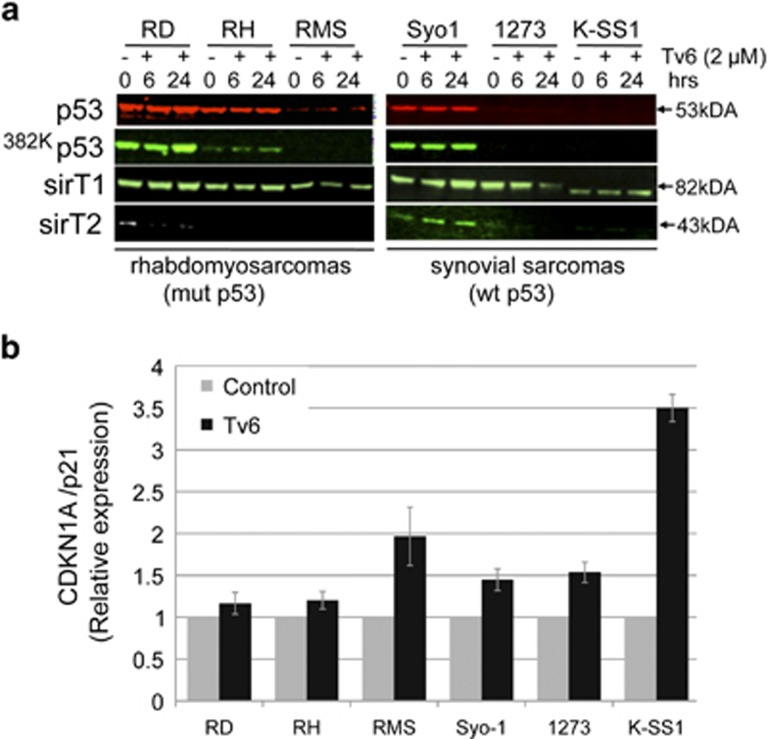
Tenovin-6 induces p21 expression without affecting p53 acetylation at lysine 382. (**a**) Expression of p53 and acetylated p53 (^Ac 382K^p53). Cells were incubated with 2 *μ*M Tv6, and total protein extracts from the cells were collected after 6 and 24 h for western blot using specific antibodies and infrared conjugates (IR-dye conjugates), and visualized simultaneously using the Odyssey system. (**b**) CDKN1A/p21 expression in Tv6-treated and control cells determined by QRT-PCR. Relative expression in relation to GAPDH is shown

Tv6 was described as an activator of wild-type p53 and as SIRT1 and SIRT2 inhibitor.^6^ We therefore investigated whether the antiproliferative effect of Tv6 in synovial sarcoma and rhabdomyosarcoma cell lines was accompanied by the stabilization and reactivation of p53 and, whether this effect was associated with sirtuin activity. We verified the p53 status in the cell lines used in this study by sequencing the exon 5–11 of the *p53* gene and we confirmed that all synovial sarcomas carry copies of wild-type *p53* gene, whereas the alveolar rhabdomyosarcomas RMS and RH carry a mutation in exon 8 of p53 (Table 1). The embryonal rhabdomysarcoma cell line RD carries a mutated *p53* gene as reported.^12^ Cell lines were exposed to Tv6 (2 *μ*M) for 6 and 24 h, and the acetylation of ^K382-^p53 was evaluated. Tv6 did not induce any change on SIRT1, SIRT2, p53 or ^K382-^p53 expression in both cells with wild-type or mutant p53. Interestingly, expression of CDKN1A/p21, a known p53 target gene, was increased in all cell lines treated with Tv6 for 6 h (Figure 3b).

Altogether, these results indicate that, in synovial sarcoma and rhabdomyosarcoma cell lines, Tv6 inhibits proliferation and induces p21 without affecting p53 or ^K382^p53 expression.

**Figure 4:**
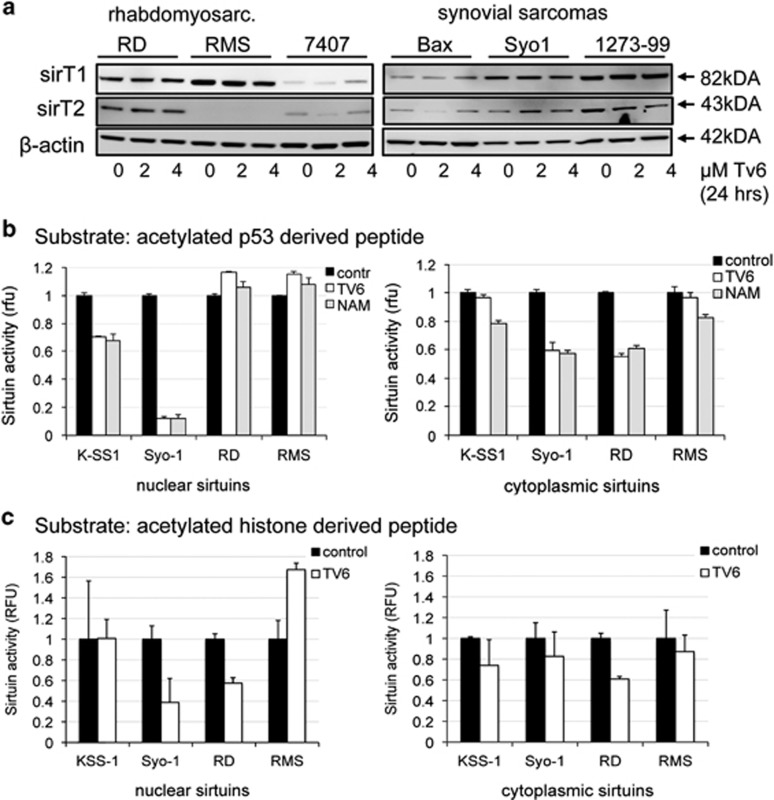
Tenovin-6 and Nicotinamide (NAM) impair sirtuin deacetylating activity. Synovial sarcoma cell lines (Syo1 and K-SS1) and rhabdomyosarcoma cell lines (RD and RMS) were exposed to tenovin-6 (2 *μ*M and 4 *μ*M) for 24 h, and protein samples were collected to determine sirtuin expression and activity. (**a**) Western blots showing the expression of SIRT1 and SIRT2 proteins before (0 h) or after exposure to TV6. (**b**) Sirtuin enzymatic activity in sarcoma cell lines exposed to sirtuin inhibitors. Nuclear- and cytoplasmic-enriched fractions were obtained from control cells and from cells exposed to Tv6 or Nicotinamide (NAM) for 6 h *in vivo*. Sirtuin activity was determined using a luciferase-based assay that measures deacetylation of a p53-derived peptide (SIRT-GLo Assay, Promega) in the presence of the HDAC inhibitor trichostatin (TSA). The assay measures sirtuin activity using a four-amino-acid acetylated long peptide (QPKK) derived from amino-acid residues 317–320 of p53. The peptide has been conjugated to an aminoluciferin reporting molecule. Experiments were repeated at least three times. (**c**) Sirtuin enzymatic activity in Tv6-treated sarcoma cell lines using an acetylated histone peptide as substrate. The experiment was performed in the same conditions as in panel **b**, with the difference that sirtuin activity was determined using a colorimetric assay that measures deacetylation of a histone-derived peptide (Epigenase Universal SIRT Activity/Inhibition Assay, Epigentek) as described in Materials and Methods

On the lack of effect of Tv6 on p53 response, in sarcoma cells, we questioned whether the enzymatic activity of sirtuins is compromised in Tv6-treated sarcoma cells. To answer this question we exposed two synovial sarcoma (Syo-1 and K-SS1) and two rhabdomyosarcoma cell lines (RMS and RD) to Tv6 for 6 h in the presence of HDAC inhibitors, and analyzed sirtuin enzymatic activity using a four-amino-acid (QPKK) acetylated peptide as substrate (see material and methods). Sarcoma cells treated with two different concentrations of Tv6 did not show any change in the overall SIRT1 or SIRT2 protein expression (Figure 4a). However, with the exception of RMS, sirtuin activity in either cytoplasmic or/and nuclear extracts was decreased in all cell lines exposed to Tv6. As control, we exposed the cells to the natural sirtuin inhibitor nicotinamide (NAM) and obtained similar results as Tv6. The inhibition of sirtuin activity by Tv6 was confirmed in an additional assay that uses an acetylated histone peptide as the sirtuin substrate (Figure 4c).

### SIRT1 and SIRT2 inhibition with tenovin-6 impairs autophagic flux

**Figure 5:**
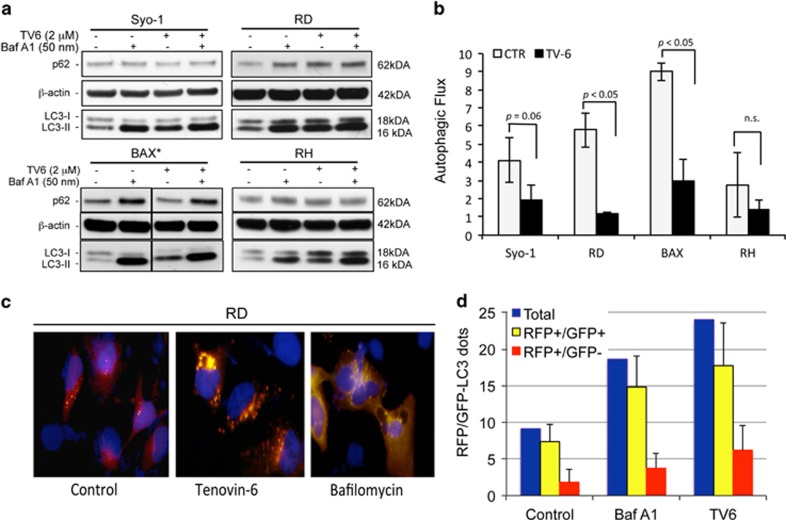
Tenovin-6 impairs autophagic flux in soft tissue sarcoma cells. (**a**) Synovial sarcoma cell lines (Syo-1 and BAX) and two rhabdomyosarcomas (RD and RMS) were exposed to tenovin-6 (TV6) at 2 *μ*M concentration (*4 *μ*M for BAX), in the presence or absence of 50 nM bafilomycin A1 (BafA1). The expression of p62 and conversion of LC3-I to its lipidated form LC3-II was determined by western blot. (**b**) The authophagic flux was determined by quantifying LC3-II in relation to *β*-actin. Autophagic flux is expressed as the ratio between LC3-II signal in the presence versus absence of Bafilomycin A1. All experiments were performed three times. (**c**) Autophagy flux determined by immunofluorescence using the tandem probe RFP-GFP-LC3. RD cells were transfected with the tandem probe, and Tv6 (2 *μ*M) was added to the cultures 24 h after transfection and incubated for additional 24 h. RD cells treated with 50 nM BafA1 for 4 h were used as control for autolysosome inhibition. Autophagosome formation is visualized as yellow speckles (RFP+/GFP+), whereas autolysosomes are visualized as red speckles (RFP+/GFP−). (**d**) Quantification of autophagosomes (yellow) and autolysosomes (red) from 10 individual cells derived from **c**. The experiment was repeated twice

It has been recently shown that Tv6 dysregulates autophagy in chronic lymphocytic leukemia (CLL) cells, suggesting that inhibition of the protective effects of authophagy induces CLL cell death following exposure to Tv6. To investigate whether a similar mechanism explains the response to Tv6 in sarcoma cells, we evaluated the autophagic flux in in two synovial sarcoma and two rhabdomyosarcoma cell lines after exposure to Tv6. First, we looked at the expression of the autophagy-associated protein LC3I and its conversion to the autophagosomal form LC3-II and observed that Tv6 induced a time-dependent accumulation of LC3-II in all cell lines indicating autophagosome accumulation (data not shown). However, as this increment could be the result of increased autophagosome biogenesis or decreased turnover of autolysosomes due to reduced lysosomal fusion and/or activity, we evaluated the expression of LC3II in cells treated with Tv6 in the presence or absence of Bafilomycin A1 (BafA1), an inhibitor of vacuolar H-ATPase (V-ATPase), an enzyme that blocks acidification of organelles such as lysosomes. As shown in Figure 5a, treatment with Tv6 at 2 *μ*M concentration for 24 h resulted in the accumulation of LC3-II in all the cell lines tested. Similarly, Baf1A induced LC3-II accumulation. However, the combination of both BafA1 and Tv6 treated did not show any additive effect on LC3-II levels. Quantitative analysis of the autophagic flux, expressed as the ratio of LC3-II signals in presence and absence of BafA1, showed a significant reduction of autophagic flux in the four sarcoma cell lines exposed to Tv6, although this was not statistically significant for RH cells. The analysis of SQSTM1 (p62), another autophagy substrate that binds LC3-II and is delivered to autolysosomes for degradation, supported the finding that Tv6 treatment has an inhibitory effect on the autophagic flux (Figure 5a).

These results were confirmed using the tandem probe RFP-GFP-LC3.^13^ Transfection of this construct into cells allows to distinguish autophagosomes (RFP+/GFP+, yellow dots) from autolysosomes (RFP+/GFP−, red dots), as GFP fluorescence is quenched at the acidic pH of the autolysosomes. The RFP-GFP-LC3 construct was transfected into the RD cell line that displayed the highest autophagy inhibition following Tv6 treatment. Cells were then exposed to 2 *μ*M Tv6 for 24 h. RD cells treated with 50 nM BafA1 for 4 h were used as control. We observed that treatment with Tv6 induced an increment in the number (and likely the size) of RFP^+^/GFP^+^ (yellow) speckles, indicating accumulation of autophagosomes. As expected, treatment with BafA1 caused a similar accumulation of yellow dots as shown in Figures 5c and d.

### Nutrient deprivation enhances the tumor antiproliferative effect of tenovin-6

**Figure 6:**
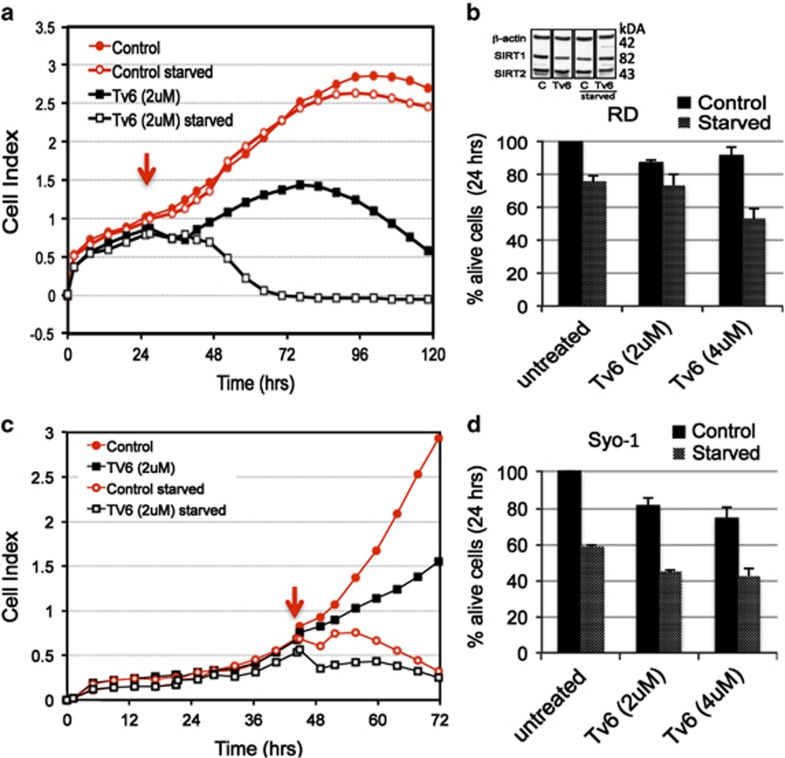
Activity of tenovin-6 in normal and in nutrient-deprived conditions. Proliferation curves of RD (**a**) and Syo-1 (**c**) cells exposed to tenovin-6 (2 *μ*M) in normal and nutrient-deprived medium (starved). Tv6 was added when cells reached exponential growth (arrow). Panels **b** and **d** show the viability of cells exposed to Tv6 in normal medium and starved conditions for 24 h measured using a colorimetric assay. See Materials and Methods

Sirtuins, in particular SIRT1, SIRT3 and SIRT6, have been implicated in the regulation of glucose and lipid metabolism during cellular stress induced by calorie restriction.^3^ With this in mind, we investigated the activity of Tv6 in nutrient-deprived conditions. Two representative sarcoma cell lines Syo-1 and RD were plated in cell culturing chambers and allowed to reach exponential growth. At this point, the medium was replaced with either normal or nutrient-deprived (glucose/essential amino-acid deficient) medium containing 2 *μ*M Tv6 or vehicle, and cell proliferation was followed in real time as long as control starved cells remained viable. Figures 6a and c show that the antitumor growth effect of Tv6 was enhanced in starving nutrient-deprived conditions in both Syo-1 and RD cell lines. However, the response curves were different. In RD cells, the deleterious effects of nutrient deprivation were evident after 96 h, whereas Syo-1 cells were highly sensitive to starving conditions and a rapid drop in cell viability was observed within the first 10 h following nutrient deprivation. Yet, the effect of Tv6 activity in normal and nutrient-deprived conditions was evident in Syo1 cells. These results were confirmed in a similar experiment where cell viability was determined at 24 h using a chromogenic assay (Figures 6b and d).

We did not observe any change in SIRT1 or SIRT2 protein expression in RD cells treated with Tv6 in normal or starvation conditions (Figure 6, RD cells).

### siRNA knockdown of SIRT1 and SIRT2 impairs rhabdomyosarcoma cell growth and LC3 II expression

**Figure 7:**
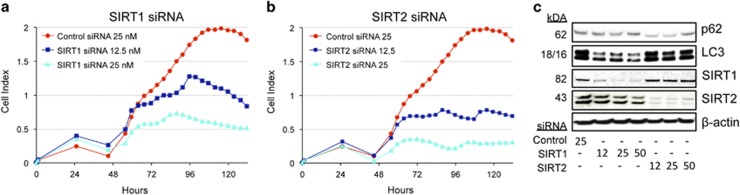
Sirt1 and Sirt2 expression is crucial for the survival of rhabdomyosarcoma cells. siRNA molecules targeting either SIRT1 (**a**) or SIRT2 (**b**) mRNA or control siRNA were reverse transfected into the rhabdomyosarcoma cell line RD at 12 and 25 nM concentrations, and cell proliferation was followed in the xCELLigence bioanalyzer for 120 h. sirT1 and sirT2, LC3II and p62 protein expression was determined by western blot using specific antibodies and is shown in **c**. 12, 25 and 50 denote nanomolar concentration siRNA

To this point, our results demonstrate that the enzymatic activity of sirtuins is compromised in soft tissue sarcoma cells exposed to the sirtuin inhibitor Tv6. To prove whether sirtuins are essential to sarcoma cell growth, we knocked down SIRT1 and SIRT2 in RD cells using a cocktail of four different siRNAs for each sirtuin to avoid off-target effects, and followed cell proliferation for 120 h. Figure 7 shows that the transcriptional silencing of both SIRT1 (Figure 7a) and SIRT2 (Figure 7b) impaired the proliferation of RD cells in a dose-dependent manner, whereas control siRNA-transfected cells proliferate up to 96 h. Interestingly, the response of RD cells to SIRT1 and SIRT2 depletion was evident at 48 h. At this time, sirtuin protein expression is clearly decreased (Figure 7c), and nutrient supply is most probably deficient in the cell culturing medium.

To investigate a possible association between SIRT1 or SIRT2 expression and autophagy, we determined the expression of the autophagy-associated proteins LC3II and p62 in SIRT1 and SIRT2 knocked down cells. Figure 7c shows that LC3 expression was decreased in SIRT1 knockdown cells, suggesting an association between SIRT1 and autophagy.

### Tenovin-6 impairs rhabdomyosarcoma xenograft growth

Mice were inoculated subcutaneously with the rhabdomyosarcoma cell line RD and allowed to form palpable xenografts before treatment with Tv6 at a concentration of 25 mg/kg/day.

**Figure 8:**
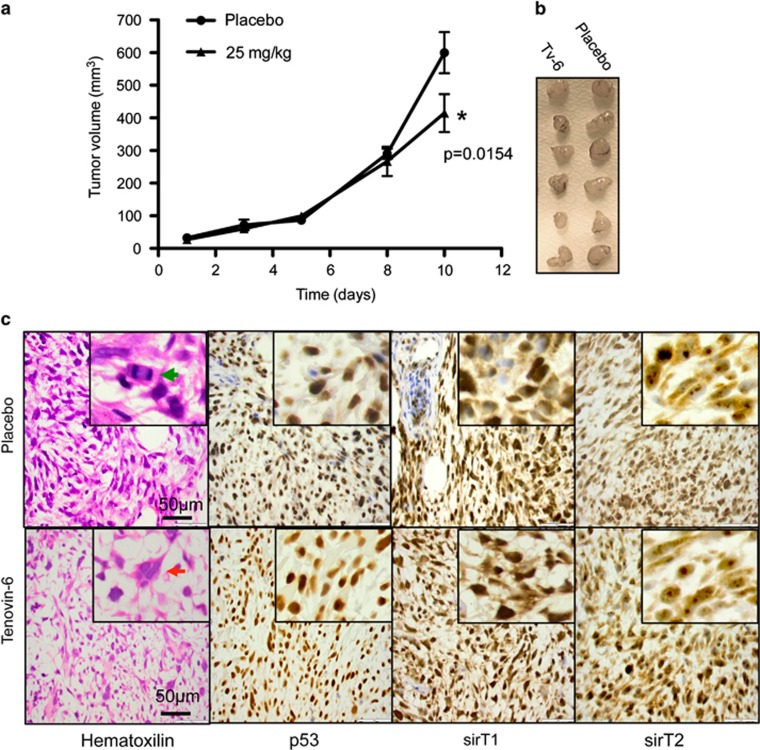
Effect of TV6 on the growth of ERMS xenografts. The embryonal rhabdomyosarcoma cell line RD was xenografted in SCID mice until tumors were palpable. Mice, eight per group, were treated with Tv6 at a dose of 25 mg/kg/day, while control group were treated with vehicle. (**a**) Tumor volume was evaluated as described in Materials and Methods. The curve shows statistically significant differences between Tv6-treated and vehicle-treated groups (*P*=0.0154, Student's *t*-test, two sided), on the last day of treatment. (**b**) Excised tumors. (**c**) Hematoxilin/eosin staining and p53, sirT1 and sirT2 immunohistochemistry of a representative sample from control and Tv6- treated RD xenografts. SIRT1 shows cytoplasmic and nuclear staining with stronger staining in the nuclear compartment. SIRT2 staining is predominantly cytoplasmic staining with clear nucleolar accumulation of the protein. Immunohistochemical staining for p53 shows homogeneous strong nuclear staining in tumor cells from control and treated mice. Green arrow: mitotic cell. Red arrow: polynuclear giant cell

Figure 8a shows that Tv6 had an inhibitory effect on the growth of rhabdomyosarcoma xenografts, with data points becoming significant on the last day of treatment (*P*=0.054, Student's *t*-test, two-sided).

Microscopically, the hematoxylin and eosin staining showed a tumor composed of pleomorphic spindle cells with macronucleoli and eosinophilic cytoplasm. Multinucleated giant tumor cells were ocasionally observed (red arrow). Frequent mitosis was easily recognized (green arrow). No differences in the composition or mitotic activity were noted between the sections prepared from mice with or without treatment (Figure 8c).

SIRT1 staining showed cytoplasmic and nuclear staining with stronger staining in the nuclear compartment. SIRT2 staining showed predominantly cytoplasmic staining with clear nucleolar accumulation of the protein. Immunohistochemical staining for p53 showed homogeneous strong nuclear staining in tumor cells from control and treated mice.

In summary, no differences were noted in the staining intensity, intracellular distribution or percentage of positively stained cells for SIRT1, SIRT2 and p53 in tenovin-6-treated or untreated control tumors (Figure 8c).

## Discussion

In the present study, we have explored the role of sirtuin expression and activity in the proliferation and survival of synovial sarcoma and rhabdomyosarcoma cell lines.

We found that SIRT1 is overexpressed in synovial sarcoma tumors and synovial sarcoma cell lines in comparison with normal mesenchymal cells. The levels of SIRT1 were also significantly higher than those of SIRT2 in almost all samples tested. Similarly, other reports have shown that SIRT1 is overexpressed in malignant epithelial tumors,^13^ breast cancer,^14^ prostate tumors,^15^ soft tissue sarcomas,^16^ cutaneous T-cell lymphomas^17^ and melanoma,^18^ and high SIRT1 expression is associated with poor prognosis in diffuse B-cell lymphomas,^19^ ovarian and breast cancers.^13,14^ The significance of SIRT1 expression in tumor oncogenesis is, however, controversial due to the dual role of this sirtuin: on one side a negative effect on p53 function^5,20^ and on the other side its involvement in the maintenance of DNA integrity. The SIRT1 knockout mouse model supports a tumor suppressor function for SIRT1,^9^ and, similar to other suppressor genes, SIRT1 is activated under cellular stress conditions, such as nutrient deprivation.

We found that the pharmacological inhibition of SIRT1 and SIRT2 with tenovin-6 effectively inhibited the proliferation of seven pediatric sarcoma cell lines at concentrations between 2 and 5 *μ*M (Table 1). As this drug inhibits SIRT1 from deacetylating p53 at K382 in several tumor cells stabilizing p53 expression and reactivating its apoptotic function,^4, 5, 6^ we hypothesized that acetylation of ^K382-^p53 could be as well compromised in Tv6-treated sarcoma cells; however, we found that the antiproliferative effect of Tv6 was independent of the *p53* gene status (wild type or mutated), ^K382-^p53 acetylation or apoptotic function. Similar results showing that Tv6 has antitumor activity independent on *p53* gene status have been reported for chronic lymphocytic leukemia^21^ and gastric cancer models.^22^

Interestingly, the expression of the the cyclin-dependent kinase inhibitor p21(cip1/waf1 CDKN1A), a *p53* target gene, was increased in all cell lines exposed to Tv6. Several studies have shown that HDAC inhibitors activate p21 expression. This has been explained by an increased acetylation of histones surrounding the p21 promoter region.^23^ A similar mechanism could explain p21 upregulation by the class III HDAC (sirtuin) inhibitors. In addition, it was recently reported that tenovin analogs promote p21 expression but fail to increase p53 levels or transcription factor activity.^6,24^

The protein levels of SIRT1 and SIRT2 remained unchanged; however, sirtuin enzymatic activity was compromised in Tv6- and nicotinamide (NAM)-treated cells. This was demonstrated using two independent enzymatic assays using either a four-amino-acid acetylated peptide derived from the p53 sequence or a histone-derived peptide. It is unlikely that the size of this peptide mimics deacetylation of full-length p53.

Seven human sirtuins have been identified acting on a widespread number of substrates. We did some attempts to identify sirtuin substrates potentially modulated by Tv6 such as *α*-tubulin, FOXO1A and histone H4 (data not shown). The *FOXO1* gene was particularly interesting since it is fused to either the *PAX3* gene or the *PAX7* gene in alveolar rhabdomyosarcoma. Moreover, cytosolic FOXO1 has been associated with autophagy induction as it is deacetylated by SIRT2.^25^ However, we did not find changes in the acetylation of FOXO1A following Tv6 treatment of rhabdomyosarcoma cells (data not shown).

An interesting finding is that the cytotoxic effect of Tv6 is associated with a decreased sirtuin deacetylase activity and with an impaired autophagic flux. The effects of Tv6 on the autophagy flux was studied by two different methods: LC3II quantification and by using a RFP/GFP LC3 tandem probe that allows the microscopic visualization of autophagosomes and autolysosomes. Both methods showed that Tv6 induces a clear accumulation of autophagosomes. Similarly, McCallum and colleagues^21^ have reported that Tv6 dysregulates autophagy in CLL cells, although the association of autophagy with sirtuin activity was not investigated in this study.

Other reports have linked SIRT1 to the regulation of authophagy such as the studies that showed that *sirT1* (−/−) mouse embryonic fibroblasts fail to activate autophagy in response to nutrient deprivation, and that SIRT1 directly interacts and deacetylates several authopagy-related proteins like Atg5, Atg7 and Atg8. The SIRT1 knockout mice undergo embryonic lethality, nevertheless the SIRT1 (−/+) mouse develops tumors when crossed with the p53 −/+ mouse.^26^ In embryonic stem cells, SIRT1 positively regulates autophagy, increases mitochondrial function and reduces oxidative stress, indicating that SIRT1 has a protective role under cellular stress conditions.^25^

Consistently, sirtuin inhibition studies with agents such as sirtinol, EX-527 and with siRNA have shown that SIRT1 inhibition impairs autophagy.^27,28^

Resveratrol, caloric restriction and oxidative stress induce autophagy and SIRT1 expression in tumor cell lines.^29^ In our study, autophagic cell death induced by sirtuin inhibition was enhanced in nutrient-deprived conditions, and the proliferation of SIRT1 and SIRT2 knockdown cells was only evident after 2 days in culture, a time point in which nutrient availability is limited in the culture media. These results support a protective role for SIRT1 in fast growing tumors in which blood supply, oxygen and nutrients become limited.

Using siRNA to knock down SIRT1 and SIRT2 expression, we demonstrated that SIRT1 and SIRT2 expression is crucial for the survival of sarcoma cells. We also showed that SIRT1-siRNA knocked out cells had a decreased expression of LC3-II, adding support to the association between SIRT1 and autophagy and suggesting that acetylation of the autophagy-related proteins such as atg 5, atg7 or atg8 could be affected as a consequence of SIRT1 inhibition with Tv6. Interestingly, p53 is both positive and negative regulator of autophagy and transactivates autophagy-associated genes in response to metabolic stress.^30,31^

*In vivo*, tenovin-6 decreased the growth of a fast growing embryonal rhabdomyosarcoma (RD) xenografts without affecting p53 or SIRT1 and SIRT2 expression. Tv6 has shown significant antitumor activity *in vivo*, alone or in combination with imatinib, in a chronic myeloid leukemia model.^32^ Possibly, the antitumor effect of Tv6 in solid tumors could be enhanced in combination with drugs inhibiting vascularization or inducing metabolic stress.

In conclusion, our study demonstrates that SIRT1 and SIRT2 expression and activity are crucial for the survival of synovial sarcomas and rhabdomyosarcomas, and that SIRT1 and SIRT2 inhibition impairs the autophagy process inducing cell death.

## Materials and Methods

### Cell lines

Synovial sarcoma cell lines: SYO-1 (provided by Dr. A. Kawai), K-SS1 (established in our lab from a monophasic SS18/SSX1 synovial sarcoma biopsy); 1273-99 (obtained from Istituto Ortopedico Rizzoli, Bologna), Bax (from Institute Cancer Research, Sutton). Rhabdomyosarcoma cell lines: RMS and RH30 are alveolar rhabdomyosarcomas, and RD is an embryonal rhabdomyosarcoma (all provided by J Shipley, Institute of Cancer Research, Sutton). All cell lines were grown as adherent cultures and were passaged in DMEM/F12 medium supplemented with 100 units/ml penicillin, 100 *μ*g/ml streptomycin (Sigma, St. Louis, MO, USA) and 10% heat-inactivated fetal bovine serum (High clone III, HyClone, Cramlington, UK).

### Cell assays to determine the activity of tenovin-6 *in vitro*

Tenovin-6 was kindly provided by Lain S. For *in vitro* assays, a 10-mM stock was prepared in dimethyl sulfoxide (DMSO) (Sigma-Aldrich, St. Louis, MO, USA), from which working solutions were freshly prepared to the desired concentrations. In all cytotoxicity assays, cells were plated and allowed to attach overnight before drug treatment.

Drug effect on cell proliferation was analyzed in real time over a period of 96–120 h using the xCELLigence cell analyzer (Roche, Basel, Switzerland) that measures electrical impedance imposed by adherent cells and is expressed as a value relative to confluence (cell index). In this assay, the number of cells per well was optimized for each cell line to get logarithmic cell growth during 96 h.

IC_50_ was determined by using WST1 assay (Roche) based on the enzymatic digestion of tetrazolium salt by viable cells. In this assay cells were plated at ~30–50% confluence depending on the growth rate of each cell and to keep control untreated cells viable (see cell pictures in Supplementary Figure 1). Percentage of viability after drug administration was calculated in relation to untreated control. Each assay was repeated at least three times.

Apoptosis was determined in sarcoma cell lines exposed to different concentrations of Tv6 during 48 h by measuring caspase 3/7 activity using a luminescence assay (ApoTox Glow, Promega, Madison, WI, USA) according to the manufacturer's instructions.

To study the activity of tenovin-6 in nutrient-deprived conditions, cells were plated in xCELLigence chambers or 96-well plates and allowed to form monolayers. Once the cells reached exponential growth phase, Tv6 was added at the desired concentration in normal media or media depleted of glucose and essential amino acids (Earle's Balances Salt Solution (EBSS-media)) containing 2% FBS. The media of untreated controls was also replaced with fresh normal medium (control) or EBSS-2%FBS (control starved cells). Cell proliferation was followed in real time until control-starved cells stopped proliferating. Cell viability was also measured using an independent colorimetric assay based on the determination of alkaline phosphatase in alive cells.

### Western blot and antibodies

Cells were seeded and incubated overnight to let them adhere. The next day cells were treated with tenovin-6 as specified in the text. Cells were then collected in loading buffer, containing 10% *β-*mercaptoethanol, sonicated, heated for 10 min at 70 °C. Cell lysates were separated by SDS-PAGE (Invitrogen, Carlsbad, CA, USA) on 4–12% gradient gels under denaturing conditions and proteins transferred onto PDVF or nitrocellulose membranes (Amersham, Buckinghamshire, UK). After blocking in 5% BSA (Sigma-Aldrich), the membranes were incubated overnight at 4 °C with the primary antibodies: p53 DO1 (Calbiochem, Darmstadt, Germany), ^Acetyl K382^p53 (Cell Signaling Technology, Danvers, MA, USA), *β*-actin, SirT1 and sirT2 (Atlas Antibodies, Stockhom, Sweden), LC3-II and p62 (Cell Signaling Technology). After a series of washes in PBS/0.1% Tween20 (PBS-T), the membranes were incubated for 1 h with the secondary antibody conjugated to horseradish peroxidase (Pierce Biotechnology, Rockford, IL, USA). Membranes were subsequently washed in PBS-T and developed using chemiluminescence western blotting detection reagents (Pierce Biotechnology).

When using the Oddisey system for western blot detection, we use nitrocellulose membranes western transfer and blocking buffer fron the manufacturer. For detection, we used infrared fluorescence IRDye secondary antibodies for the simultaneous detection of p53 and acetylated p53.

### QRT-PCR

Total RNA was isolated using RNeasy (Qiagen, Hilden, Germany). In brief, 500 ng of total RNA was reverse transcribed to cDNA, using random hexameres and SuperScript II reverse transcriptase (Invitrogen, Life Technologies, Carlsbad, CA, USA), according to the manufacturer's instructions. Gene expression analysis was performed in quadruplicates of each sample in a total volume of 20 *μ*l using TaqMan probes: hs01009005(SIRT1) and hs00247263(SIRT2) (TaqMan, Life Technologies). Each assay was repeated three times.

### Sirtuin activity in nuclear and cytoplasmic extracts from tenovin-6-treated cells

For the quantitation of sirtuin activity, cells (~3 × 10^6^) were cultured to 80% confluence and starved in serum-free media overnight before exposure to 4 *μ*M TV6 in medium containing FBS, or only medium with FBS (control untreated cells) for 6 h. The cells were then scraped and collected in hypotonic buffer (containing 10mM Tris Ph 7.5, 10 mM NaCl and 1.5 mM MgCl_2_, protease and HDAC inhibitors), cryo-lyzed with three cycles of freezing (liquid nitrogen), and thawed and centrifuged at 8000 r.p.m. for 5 min to separate the cytoplasmic from the nuclear fractions. The nuclei containing pellets were washed in hypotonic buffer, resuspended in NP40 buffer (150 mM NaCl, 1% NP40, 50 mM Tris pH 8) and sonicated. Protein concentration in the subcellular extracts was determined before the determination of sirtuin activity using a standard Bradford method.

The enzymatic activity of sirtuins was quantitated in three independent experiments using two independent methods: SIRT-Glo Assay (Promega), which is a luminescent assay that measures the relative activity of sirtuins from purified enzyme sources. The system uses as a substrate an acetylated four-amino-acid peptide (QPKK) derived from amino-acid residues 317–320 of p53. The peptide has been conjugated to an aminoluciferin reporting molecule; sirtuin activity is therefore assessed by luminescence.

Sirtuin activity was also determined using a fluorometric assay Epigenase Universal SIRT Activity/Inhibition Assay (Epigentek, Farmingdale, NY, USA) that has been optimized for measuring sirtuin activity from nuclear extracts using a histone-derived substrate.

### Authophagy studies

Autophagy flux was examined by quantifying the conversion of the cytosolic LC3I to the autophagosome membrane bound LC3II in the presence of bafilomycinA1 and by quantifying the number of autophagosomes and autolysosomes in cells transfected with the tandem RFP/GFP-LC3II vector and exposed to tenovin-6 or BafA1.

Cells were plated in their standard medium and allowed to adhere overnight before the addition of tenovin-6 (2 μM) for 24 h. Bafilomycin 1A at 50 nM concentration (Sigma B1793) was added to the cell cultures 4 h before the end point of the experiment.

The protein lysates were sonicated and resolved in 4–12% polyacrilamide gels (Invitrogen), and western blot was performed to detect the expression of LC3I/LC3II and p62 using the following antibodies: p62 (BD Transduction Laboratories, Franklin Lakes, NJ, USA, 610833), LC3 (Cell Signaling Technology, 2775) and *β*-actin (Sigma, A5441). HRP-conjugated anti-rabbit (NA934V), and anti-mouse (NXA931) antibodies, ECL system (RPN2106) were from GE Healthcare (Pittsburgh, PA, USA).

A tandem RFP/GFP fluorescent-tagged LC3 plasmid was used to assess autophagosome maturation process as previously described.^33^ RD cells plated in round coverslips were transfected with 1 *μ*g of tandem RFP/GFP plasmid and exposed to 2 *μ*M tenovin-6, 24 h after transfection. BafA1 was added 4 h before the end point of the experiment (24 h after Tv6). The coverslips were removed from the medium,washed three times with PBS and fixed in freezer cold ethanol :acetone 1 : 1 for 15 min, dried and mounted in DAPI solution before visualization in a fluorescent light microscope.

### SIRT1 and SIRT2 siRNA transfections

RD cells were reverse transfected with SIRT1-siRNA or SIRT2-siRNA molecules each consisting of four individual siRNAs targeting different sirtuin regions to avoid off-target effects (ON-TARGETplus Human SIRT1 (23411) siRNA and Human SIRT2 (22933)—SMARTpool) using DharmaFECT1 transfection reagent. Control cells were transfected with siGENOME non-targeting siRNA pool. We followed the manufacturer's recommendation for transfection optimization.

### Activity of tenovin-6 *in vivo*

Animal studies were approved by the ethics committee Stockholms Norra Djurförsöksetiska Nämnd to B.B. (N80/11).

Fox Chase SCID mouse, CB17/lcr-*Prkdc*SCID/lcrCrl (~8 weeks old, 20–30 g) were inoculated subcutaneously in the right flank with 3 × 10^6^ RD cells resuspended in 100 *μ*l matrigel (BD Biosciences, San Jose, CA, USA). Mice, (eight per group) were treated with either tenovin-6 (Tv6) or placebo once tumors became palpable (approximately day 8 after inoculation). Both drug and placebo were delivered by subcutaneous introduction of a 21-day release pellet. The slow-release pellets maintained steady drug levels of 25 mg/kg/day during the 10-day course of the experiment.

Tumor dimensions were recorded twice per week. At the end point of the experiment, mice were killed by cervical dislocation, and each tumor was excised and fixed in formalin. Tumor volumes were calculated using the equation (*l*°—*w*2)*/*2, where *l* and *w* represent the largest and smallest dimensions at each measurement. The effect of drug treatment on tumor growth was analyzed statistically using two-sided Student's *t-*test.

Immunohistochemistry of the tumors was performed using standard methods using the following antibodies: p53 (DO-1, Calbiochem) sirT1 (Atlas Ab, HPA 052351) and sirT2 (Atlas Ab, HPA011165).

## Figures and Tables

**Table 1:** Genetic markers, p53 status and histology of soft tissue sarcoma cell lines

**Sarcoma subtype**	**Cell line**	**Genetic arrangement**	***P53*****gene status**	**Tv6 IC**_**50**_
SS	K-SS1	SS18/SSX1	wt	2.7
	SYO-1	SS18/SSX2	wt	5.5
	BAX	SS18/SSX2	wt	3.6
	1273/99*	SS18/SSX2	wt	4
RMS	RMS (a)	PAX3/7-FOXO1	Mut	2.3
	RH30 (a)	PAX3/7-FOXO1	Mut	1
	RD (e)	22q-	Mut	3.5

Abbreviations: RMS, rhabdomyosarcomas; SS, synovial sarcomas. Alveolar rhabdomyosarcoma (a); embryonal rhabdomyosarcoma (e) and SV-40 transformed (*). The fusion gene type and the status of the *p53* gene was determined using RT-PCR and dye terminator sequencing. IC_50_ were determined by exposing the cells to different concentrations of tenovin-6 and determining viability after 48 h using the Wst-1 assay (Supplementary Figure 1).

## Abbreviations

SIRT1: sirtuin 1
SIRT2: sirtuin 2
HDAC: histone deacetylase
NAD+: nicotinamide adenine dinucleotide (oxidized form)
ADP: adenosine diphosphate
LC3: Microtubule-associated protein 1A/1B-light chain 3
Tv6: tenovin 6
DMSO: dimethyl sulfoxide
SS: synovial sarcoma
RMS: rhabdomyosarcoma
