# Supplementary figures and images for: SIRT1 and SIRT2 inhibition impairs pediatric soft tissue sarcoma growth
Source: Cell Death Dis. 2014 Oct 23;5(10):e1483–. doi: 10.1038/cddis.2014.385 (PMC4237232; doi:10.1038/cddis.2014.385)

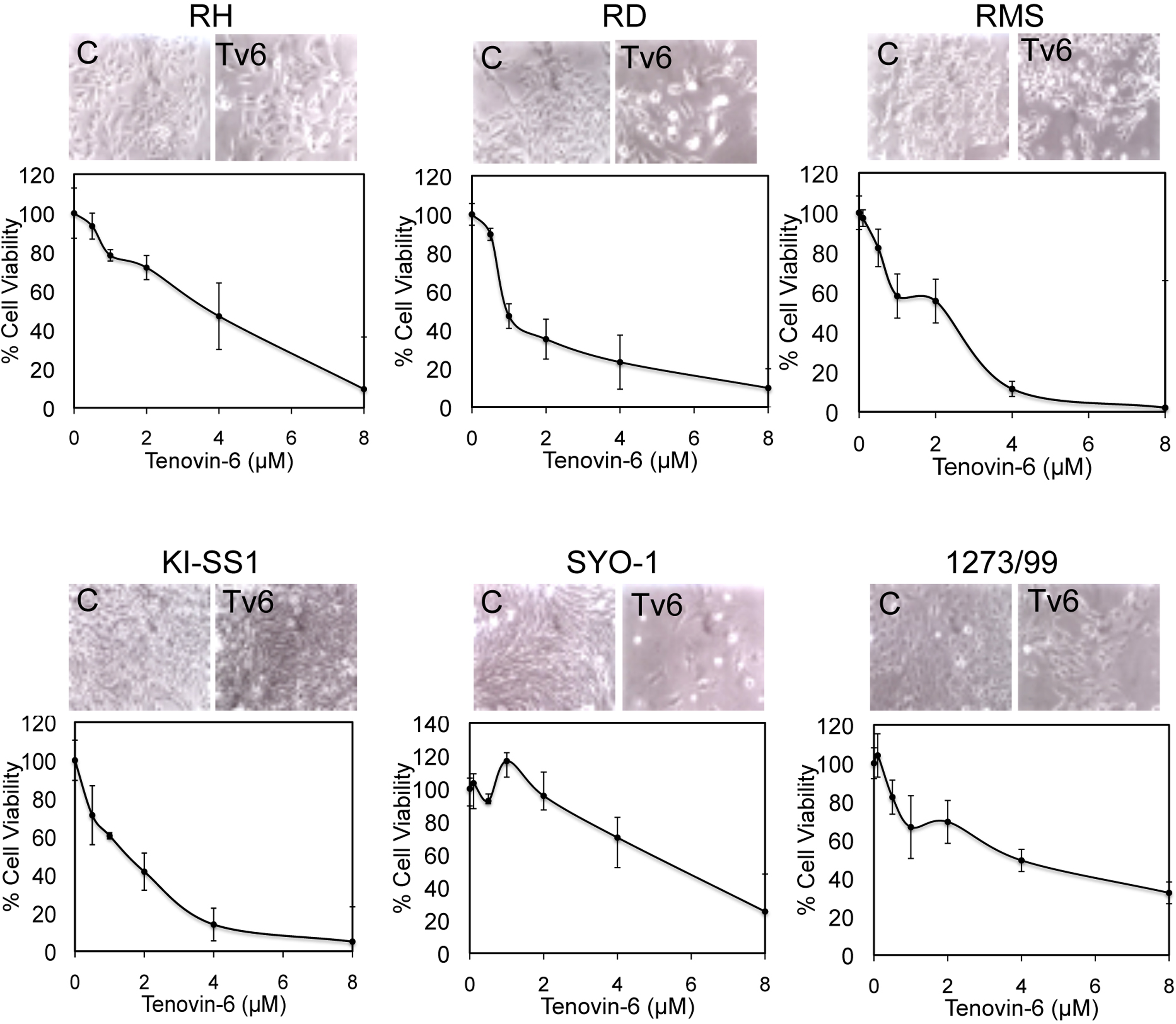

Supplement: Supplementary Figure 1 [file cddis2014385x1.tif]
