# Supplementary material for: SIRT1 and SIRT2 inhibition impairs pediatric soft tissue sarcoma growth
Source: Cell Death Dis. 2014 Oct 23;5(10):e1483–. doi: 10.1038/cddis.2014.385 (PMC4237232; doi:10.1038/cddis.2014.385)
Supplement: Supplementary Figure 1 Legend [file cddis2014385x2.doc]

**Supplement Figure 1**

*Dose dependent effect of tenovin 6 on the proliferation of rhabdomyosarcomas and synovial sarcoma cell lines.*

Cells were exposed to different concentrations of tenovin-6 for 48 hrs and proliferation was determined using the Wst-1 assay (Roche). Microscopic photographs of each cell line treated with tenovin-6 at the end point of the assay are shown on the top of each growth curve.

Rhabdomyosarcomas: RD, RH, RMS. Synovial sarcomas (KI-SS1, SYO-1 and

1273/99).
